# ICU patients with infectious complications after abdominopelvic surgery: Is thoracic CT in addition to abdominal CT helpful?

**DOI:** 10.1186/s13613-023-01104-1

**Published:** 2023-02-10

**Authors:** Heiner Nebelung, Natalie Wotschel, Hanns-Christoph Held, Johanna Kirchberg, Jürgen Weitz, Christoph Georg Radosa, Michael Laniado, Ralf-Thorsten Hoffmann, Verena Plodeck

**Affiliations:** 1 Institute and Polyclinic for Diagnostic and Interventional Radiology, University Hospital Carl Gustav Carus Dresden at the Technical University Dresden, Fetscherstr. 74, 01307 Dresden, Germany; 2 Department of Visceral, Thoracic and Vascular Surgery, University Hospital Carl Gustav Carus Dresden at the Technical University Dresden, Fetscherstr. 74, 01307 Dresden, Germany; 3 National Center for Tumor Diseases (NCT/UCC), Dresden, Germany; 4 German Cancer Research Center (DKFZ), Heidelberg, Germany; 5 Helmholtz-Zentrum Dresden-Rossendorf (HZDR), Dresden, Germany

**Keywords:** Diagnostic imaging, Multidetector computed tomography, Intensive care units, Surgery, Infections

## Abstract

**Background:**

The aim of this study was to assess the usefulness of adding thoracic CT to abdominal CT in intensive care unit (ICU) patients with signs of infection after abdominopelvic surgery.

**Methods:**

143 thoracoabdominal CTs of ICU patients with signs of infection after abdominopelvic surgery were retrospectively reviewed for thoracic pathologies. It was determined if pathologic findings were visible only on thoracic CT above the diaphragmatic dome or also on abdominal CT up to the diaphragmatic dome. All thoracic pathologies visible only above the diaphragmatic dome were retrospectively analyzed by an ICU physician in terms of clinical relevance. Diagnostic and therapeutic efficacy of thoracic CT were assessed with regard to an infectious focus and to other pathologic findings.

**Results:**

297 pathologic thoracic findings were recorded. 26 of the 297 findings could only be detected on images obtained above the diaphragmatic dome (in 23 of 143 CTs). A change in patient management was initiated due to only one of the 26 supradiaphragmatic findings. Diagnostic efficacy of thoracic CT in addition to abdominal CT to identify an infectious focus was 3.5% (95%-CI: 0.5–6.5%) and therapeutic efficacy was 0.7% (95%-CI: 0–2.1%). With regard to all pathologic thoracic findings, diagnostic efficacy was 16.1% (95%-CI: 10.1–22.1%) and therapeutic efficacy remained at 0.7%.

**Conclusions:**

Additional thoracic CT to detect an infectious focus in ICU patients after abdominopelvic surgery leads to identification of the focus in only 3.5% and to changes in patient management in only 0.7%. Other relevant findings are more common (16.1%), but very rarely affect patient management.

## Background

Infectious complications after surgery are common. Around 11% of patients develop nosocomial infections [1], with postoperative pneumonia affecting around 5% of patients [2] and up to 40% of patients developing surgical site infections, depending on the type of surgery [3]. Severe postoperative infectious complications, such as sepsis, are also not uncommon with an overall incidence of 1.84% [4]. If sepsis occurs after surgery, the infectious focus involves the abdominal cavity in 66% of patients [5]. In a cohort of septic patients with re-laparotomy after abdominal surgery, CT was found to play a significant role in identifying the septic focus [6].

Imaging generally plays an important role in the management of patients in intensive care units (ICUs). The recently published American College of Radiology Appropriateness Criteria® for Intensive Care Unit Patients state that portable chest radiography (CXR) is still the most commonly used imaging modality in ICU patients [7]. Extensive research has shown not only the usefulness of CXR but also the potential for overuse and a meta-analysis found no harm associated with a restrictive strategy [7–10].

There has been little research on the topic of thoracic CT in ICU patients, especially in recent years, but the number of CT scans has been ever increasing over the years [11–13]. An article by Dorenbeck et al. in 2002 evaluated the usefulness of thoracic CT in general ICU patients in comparison to CXR and concluded that CT resulted in a high number of additional diagnoses, another study by Miller et al. in 1998 stated that CT is useful in selected patients [14, 15].

In our center, the standard of care is to liberally perform thoracic CT in addition to simultaneous abdominal CT in patients with signs of infection after abdominopelvic surgery. The advantage of this approach is the possibility of a whole-body overview of potential infectious foci in critically ill patients. This avoids repeated, potentially detrimental patient transfers with accompanying ICU personnel and preserves critical resources. On the other hand, unnecessary CT imaging is a rising concern and should be avoided due to increased exposure of patients to ionizing radiation as well as increasing costs.

To the best of our knowledge, no study thus far has assessed the usefulness of additional thoracic CT in ICU patients with infectious complications after abdominopelvic surgery. Thus, the aim of this study was to determine the value of thoracic CT in this setting.

## Methods

### Study population and baseline characteristics

**Fig.1:**
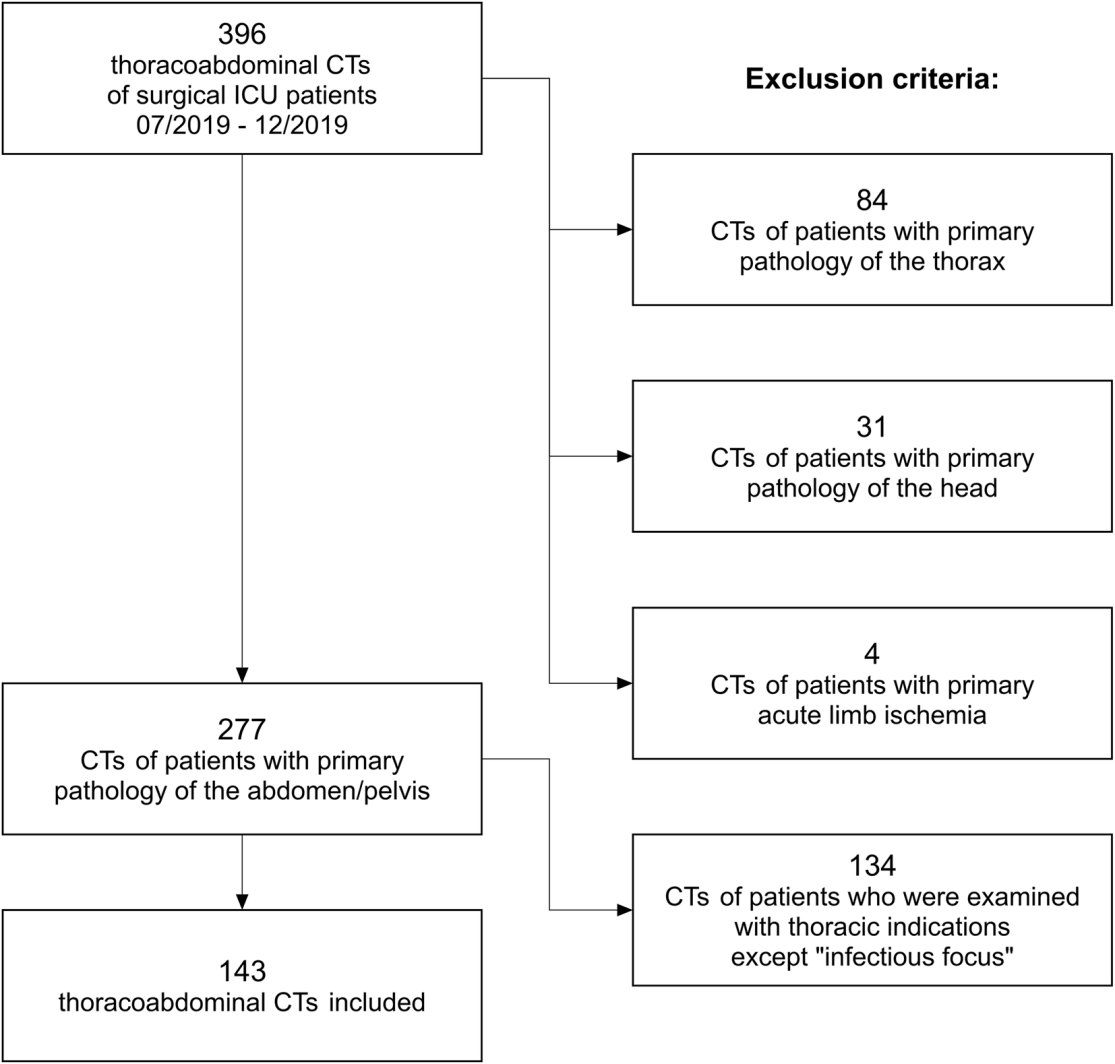
Study population and exclusion criteria

Approval by the local ethics committee was granted for this study, which was performed in accordance with the ethical standards as laid down in the 1964 Declaration of Helsinki and its later amendments. Patients, who were examined in non-emergency situations and who were able to, had given written informed consent to CT. All CTs had been performed within the scope of clinical routine. Clinical data and imaging of 180 surgical ICU patients (51 women, 129 men), who had received postoperative thoracoabdominal CT scans at our institution between July 2019 and December 2019, were retrospectively reviewed. Patients were referred for CT with clinical signs of infection, e.g., fever, elevated leukocytes, and C-reactive protein. Some patients received more than one CT; thus, a total of 396 CTs was reviewed. 119 CTs were excluded, because the patients had non-abdominopelvic primary pathologies (for details see Fig. 1). Out of the remaining 277 CTs, 134 CTs were excluded, because thoracic CT was performed for reasons other than suspected infectious focus. Examples are suspected pulmonary artery embolism, suspected thoracic hemorrhage, and follow-up of known pathologies, like thoracic abscess, pleural empyema, hemothorax, or pneumonia. The remaining 143 CTs were included in this study (99 CTs of 50 male patients and 44 CTs of 22 female patients). The mean age of patients was 62.5 years (± 14.5). At the time of imaging, mean value of leukocytes was 16.40 (± 9.48) GPt/L (reference value 3.8–9.8 GPt/L) and of C-reactive protein was 155.84 (± 101.75) mg/L (reference value < 5.0 mg/L). In 72 CTs, the patients were mechanically ventilated. The primary diagnoses and surgical procedures are summarized in Table 1.

**Table 1:** Primary diagnoses and surgical procedures of our study population

Primary diagnosis	Surgical procedure	*n*
Acute cholecystitis	Cholecystectomy	2
Chronic pancreatitis	Pylorus-preserving pancreaticoduodenectomy	2
	Duodenum-preserving pancreatic head resection	2
	Whipple's procedure	1
	Pancreatectomy	1
Diabetes mellitus type 1	Pancreas transplant	1
Pancreatic cancer	Pancreatectomy	1
	Pylorus-preserving pancreaticoduodenectomy	3
	Left pancreatic resection	1
	Multivisceral resection	1
Main duct intraductal papillary mucinous neoplasm	Pancreatectomy	1
Benign tumor of the papilla	Pylorus-preserving pancreaticoduodenectomy	1
Klatskin tumor	Pylorus-preserving pancreaticoduodenectomy	1
	Whipple's procedure	1
	Left hemihepatectomy	1
Cholangiocellular carcinoma	Right hemihepatectomy	1
	Extended right hemihepatectomy	2
Cholecystic myosarcoma	Right hemihepatectomy + cholecystectomy	1
Caroli's syndrome	Left hemihepatectomy	1
Hepatic metastases of rectal cancer	Right hemihepatectomy	1
Gastric cancer	Gastrectomy	3
	Subtotal gastrectomy	1
Recurrent gastric cancer	Pylorus-preserving pancreaticoduodenectomy	1
Esophageal cancer	Esophagectomy	1
Colon cancer	Peritonectomy + HIPEC	1
Rectal cancer	Rectal resection	1
	Rectal exstirpation	1
Recurrent rectal cancer	Pelvic exenteration	1
Recurrent chordoma	Pelvic exenteration	1
Peritoneal fibrosarcoma	Multivisceral resection	1
Peritoneal carcinosis (endometrial cancer)	Multivisceral resection	1
Colitis ulcerosa	Proctocolectomy	1
Acute abdomen	Explorative laparotomy	3
Mechanical ileus	Partial small bowel resection	1
Incarcerated incisional hernia	Hernia repair	1
Ogilvie syndrome	Subtotal colectomy	1
Sigmoid perforation	Sigmoid resection	1
Duodenal ulcer	Ulcer excision and repair	1
Upper gastrointestinal bleed	Explorative laparotomy	1
	Partial small bowel resection	1
	Pancreatectomy	2
Lower gastrointestinal bleed	Explorative laparotomy	2
Bleed from right hepatic artery	Evacuation of hematoma	1
Retroperitoneal hematoma	Embolization of lumbal artery	1
Mesenteric ischemia	Explorative laparotomy	2
	Total colectomy	2
	Subtotal colectomy	1
	Right hemicolectomy	1
	Right hemicolectomy + partial small bowel resection	1
	Left hemicolectomy + partial small bowel resection	1
Abdominal aortic aneurysm	Endovascular aortic repair	1
Infection of iliac bypass	Explantation of bypass	1
Gluteal/perianal ulcers	Debridement	4
Retroperitoneal abscess	Abscess drainage	1
Inguinal infected seroma	Wound revision	1

### Image interpretation

**Fig. 2:**
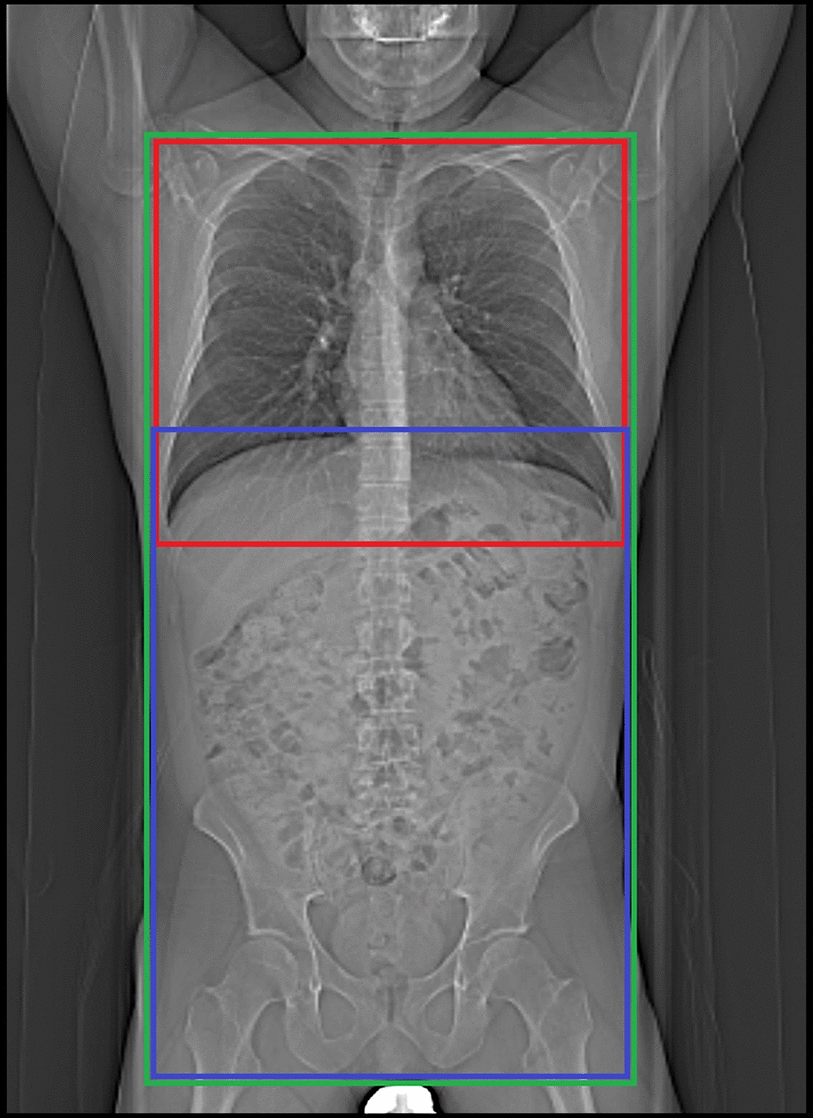
CT planning scout. Green: scout for thoracoabdominal CT. Red: examined region in thoracic CT alone. Blue: examined region in abdominal CT alone

The 143 CTs were reviewed independently by three radiologists with fifteen, three, and one year(s) of experience in CT imaging. They looked for thoracic pathologies and recorded, if they were visible only above an imaginary plane at the tip of the diaphragm or if they were visible below as well (Fig. 2). All occurring pathologies are shown in Table 2. For all thoracic pathologies, which were visible only above the diaphragmatic dome, an intensive care physician (> 15 years of experience) determined retrospectively, if they were clinically relevant. Therefore, he searched our hospital information system for changes in patient management due to the pathologic findings on CT (only above the diaphragmatic dome).

### Data analysis

To assess the usefulness of thoracic CT we employed the concept developed by Fryback and Thornbury, which defines six levels of efficacy. Efficacy of diagnostic imaging is defined as its contribution to the patient management process.

Level 1 addresses technical efficacy, level 2 the yield of abnormal or normal diagnoses in a case series, as well as diagnostic accuracy, sensitivity, and specificity associated with interpretation of the images. Level 3 focuses on whether the information results in change in the referring physician’s diagnostic thinking. Level 4 efficacy concerns possible effects on the patient management plan. Level 5 focuses on patients’ outcome and level 6 deals with societal efficacy addressing benefits and costs [16].

We determined diagnostic efficacy of thoracic CT imaging by calculating the proportion of CTs with pathologic thoracic findings visible only above diaphragmatic dome out of all performed CTs. In the next step, we determined therapeutic efficacy by calculating the proportion of CTs with pathologic thoracic findings visible only above the diaphragmatic dome that affected patient management out of all performed CTs.

Effective doses (in mSv) were calculated by multiplying the dose length products, which were provided by the scanner, with a conversion factor of 18 μSv/mGycm, as recommended by Huda et al. [17].

## Results

**Fig. 3:**
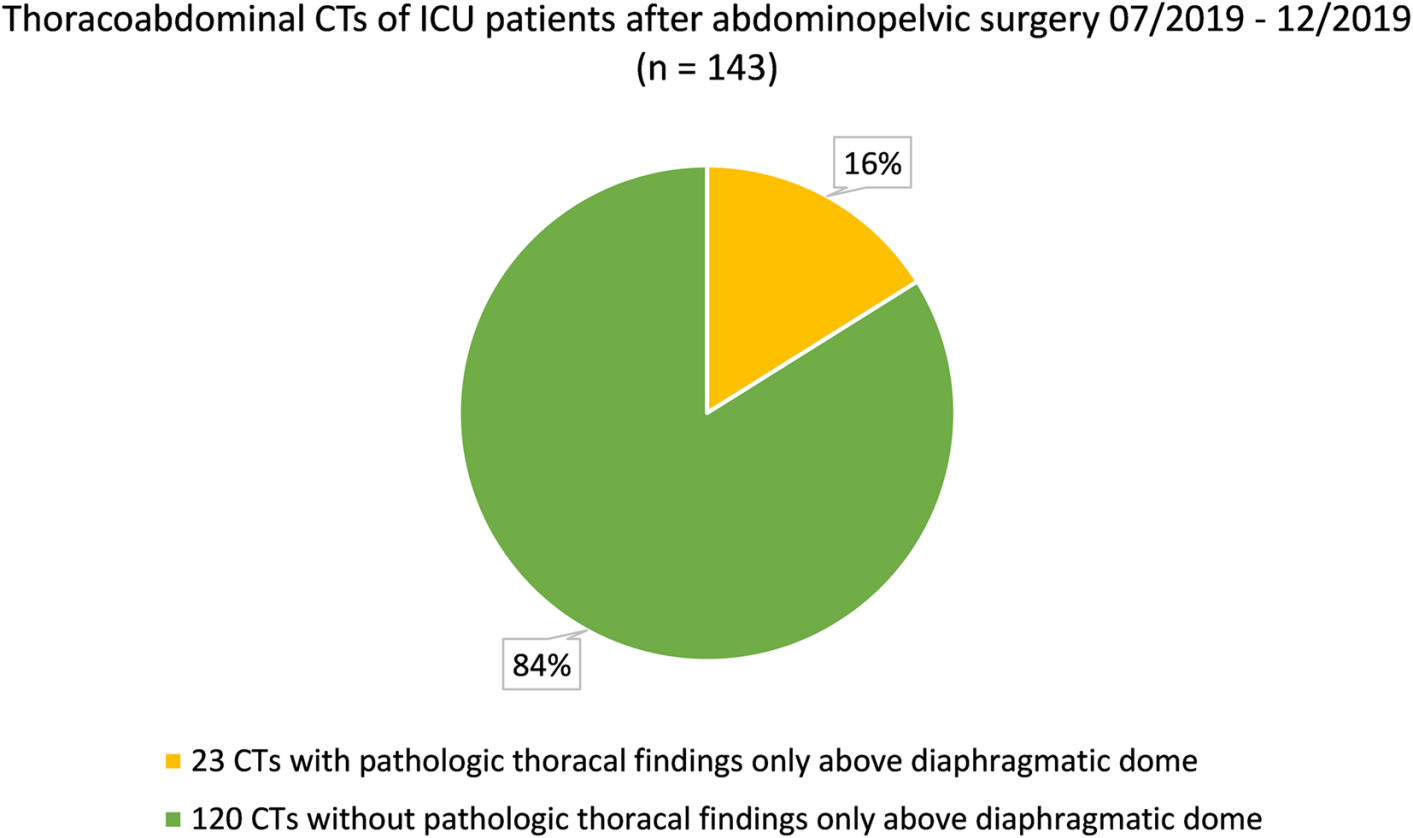
Thoracoabdominal CTs of ICU patients after abdominopelvic surgery

In 143 CTs we found a total of 297 thoracic pathologies (median 2; IQR 1), see Table 2. More than two-thirds were pleural effusions and compression atelectases. In 29.4% of the CTs, patchy consolidations of the lungs compatible with the imaging diagnosis of pneumonia were found. In most cases, pathologic thoracic findings were visible below diaphragmatic dome. Only in 23 of 143 CTs we found thoracic pathologies, which were visible only above diaphragmatic dome (Fig. 3), so overall diagnostic efficacy of dedicated thoracic CT as part of the imaging protocol was 16.1% (95%-CI: 10.1–22.1%).

**Table 2:** Pathologic thoracic findings in thoracoabdominal CT

Pathologic findings	Thoracal		Visible only above diaphragmatic dome		Clinically relevant	
		% on all 143 CTs		% on all 143 CTs		% on all 143 CTs
Pneumonic infiltrate	42	29.4%	5/42	3.5%	1/5	0.7%
Pulmonary congestion	21	14.7%	8/21	5.6%	0/8	0%
Catheter-associated thrombosis	7	4.9%	7/7	4.9%	0/7	0%
Endotracheal tube malposition	3	2.1%	3/3	2.1%	0/3	0%
Chest tube malposition	1	0.7%	1/1	0.7%	0/1	0%
Pulmonary artery embolism	1	0.7%	1/1	0.7%	0/1	0%
Pulmonary nodule	2	1.4%	1/2	0.7%	0/1	0%
Pleural effusion	131	91.6%	0/131	0%	N/A	N/A
Dystelectasis	79	55.2%	0/79	0%	N/A	N/A
Pericardial effusion	10	7.0%	0/10	0%	N/A	N/A

**Fig. 4:**
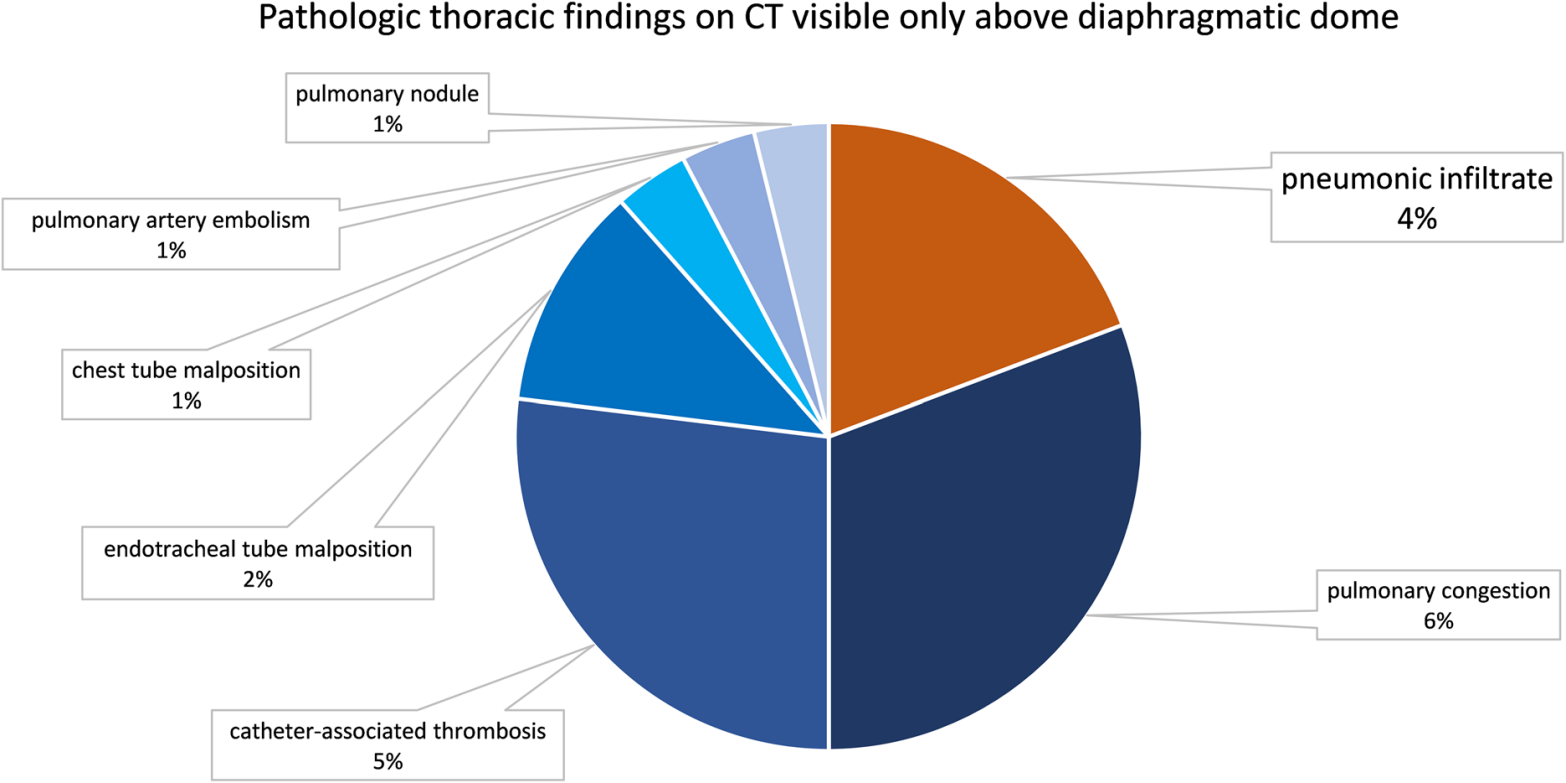
Pathologic thoracic findings on CT visible only above diaphragmatic dome

**Fig. 5:**
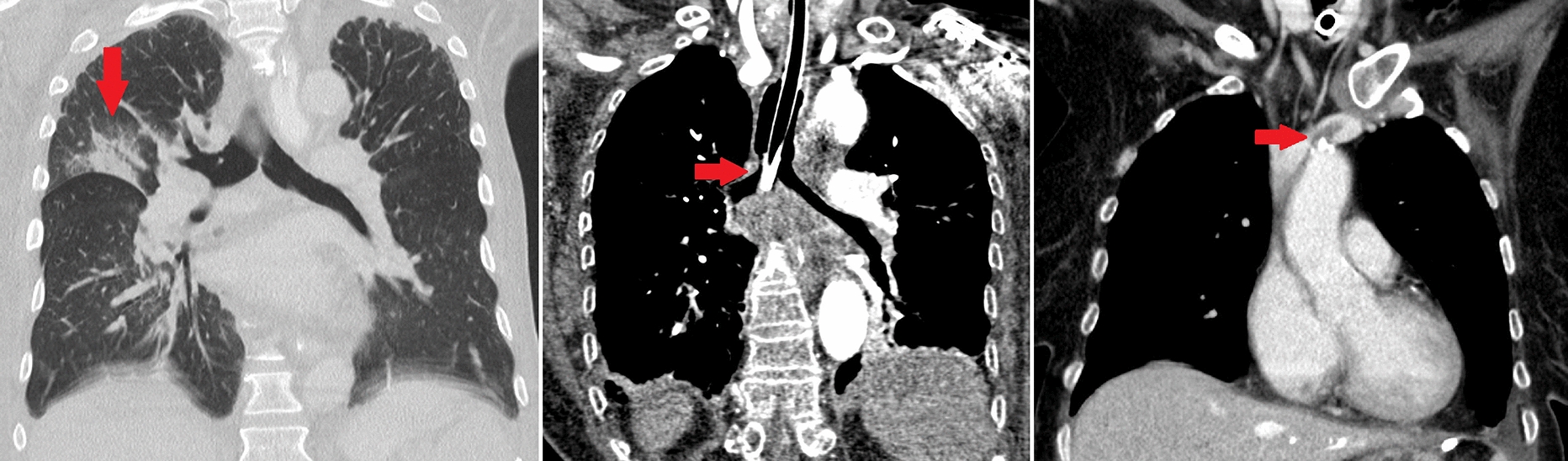
Examples of imaging findings on thoracic CT above the diaphragmatic dome. Left: Coronal contrast enhanced CT showing pneumonic infiltrate in the right upper lobe. Middle: Coronal contrast enhanced CT showing endotracheal tube malposition in the right main bronchus. Right: Coronal contrast enhanced CT showing catheter-associated thrombosis in the left brachiocephalic vein

In five cases (3.5%) we found pneumonic infiltrates, which were visible only above diaphragmatic dome (Fig. 4 and 5). There were no other findings above the diaphragmatic dome, which could be reported as an infectious focus, so the diagnostic efficacy of thoracic imaging with regard to an infectious focus was 3.5% (95%-CI: 0.5–6.5%).

In one of these five cases, antibiotic therapy was initiated due to the imaging diagnosis of pneumonic infiltrate. In the other four cases, there was no change in patient management due to the reported pulmonary infiltrate on CT, so the therapeutic efficacy of thoracic imaging with regard to an infectious focus was 0.7% (95%-CI: 0–2.1%).

There were some other pathologic findings only visible above the diaphragmatic dome (Fig. 4). In one case (0.7%) we could not exclude peripheral pulmonary artery embolism, but this did not influence patient management. In seven cases (4.9%) we found catheter-associated thromboses (Fig. 5), which in no case led to changes in patients’ management, since all patients already received anticoagulation. In three cases (2.1%) we found endotracheal tube malposition in the right main bronchus (Fig. 5) and in one case (0.7%) we found chest tube malposition within the soft tissues of the chest wall. These findings were already visible and reported on previous chest X-ray imaging. In one case (0.7%), there was a pulmonary nodule above the diaphragmatic dome. This was a known metastasis of colorectal cancer, which was equal in size (6 mm) compared to the last staging CT six weeks prior. In eight cases (5.6%) pulmonary congestion was found only above the diaphragmatic dome. Pulmonary congestion was very mild in all cases, in five of these cases it was already known prior to CT and treatment had already been initiated. In the remaining three cases fluid overload was not considered clinically relevant (in accordance with the mild extent on CT), and therefore, no change to patient management was made. Overall, therapeutic efficacy of thoracic imaging exceeding the primary goal of identifying an infectious focus was 0%.

The mean dose length product was 1191.8 (± 688.5) mGycm. The corresponding mean effective doses was 21.45 (± 12.39) mSv.

## Discussion

To identify an infectious focus in ICU patients after abdominopelvic surgery, diagnostic efficacy of thoracic CT in addition to abdominal CT was 3.5% and therapeutic efficacy was 0.7%, signifying that we could identify an infectious focus in 3.5% of the additional thoracic CTs with an effect on patient management in 0.7%. With regard to all pathologic thoracic findings, diagnostic efficacy was 16.1% and therapeutic efficacy remained 0.7%, since no other pathologic findings affected patient management.

Whereas on the use of CXR in ICU extensive research has been published, there is comparatively little data on the use of chest CT in this setting.

An article by Dorenbeck et al. in 2002 evaluated the usefulness of thoracic CT in comparison to CXR and concluded that CT resulted in a high number of additional diagnoses, with therapeutic consequences in around half of the 558 CT studies [14]. This study included patients with a variety of primary diagnoses on an anesthetist-led ICU. In total, 56% of the study population suffered from known primary or secondary pulmonary diseases and only 35% of CTs were requested to identify a septic focus. 65% of indications were pulmonary pathologies, like deteriorating gas exchange, possible misplacement of thoracic drain, and pulmonary embolism. This plausibly explains why in our study we found markedly lower numbers of previously unknown pulmonary diagnoses as well as less therapeutic consequences. Another reason could be that we assessed if pathologies were only visible on thoracic CT (above the diaphragmatic dome) or also on abdominal CT (including the diaphragmatic domes and lower lungs), which reduced diagnostic efficacy to 16.1%. The most common findings (57.7%) in the study of Dorenbeck et al. were dys-/atelectases, pneumonic infiltrates, and pleural effusions, which is in keeping with our results.

Another study by Miller et al. in 1998 included 85 patients/108 thoracic CTs in patients on a surgical (55/65%), medical, or cardiac ICU [14, 15]. 92% of all CTs were requested by thoracic surgeons, cardiac surgeons, or pulmonary physicians, suggesting that the majority of patients suffered from primary thoracic pathologies, although the primary diagnosis is not mentioned in the article. CT findings were compared with CXR and it was demonstrated that CT showed at least one new clinically important finding in 30%, which led to a change in patient management in 22%. The higher numbers compared to our study are likely due to the different study populations and our approach of differentiating between pathologies below/above the diaphragmatic dome. There have been few studies on the frequency of incidental findings on CT in ICU patients. A study by Schramm et al. found that thromboses were a common incidental finding, which is in keeping with our results [18].

In times of ever-increasing medical radiation exposure, indications for CT scans should be critically assessed on a per-case basis. Radiation exposure due to thoracoabdominal CT scans in our study population was very heterogeneous due to varying combinations of contrast phases and exam protocols. The mean dose length product was 1191.8 (± 688.5) mGycm. The corresponding mean effective doses was 21.45 (± 12.39) mSv. The diagnostic reference level (giving an indication of the expected radiation dose received by an average-sized patient undergoing an imaging procedure) for thoracoabdominal CT scans performed with one contrast phase is 1000 mGycm or 16 mSv, for abdominal CT scans 700 mGycm or 11.4 mSv [19]. These reference values cannot be easily transferred to our patient population due to the complexity of ICU patients and therefore frequently extensive necessary CT protocols, but it is still obvious that the addition of thoracic CT to abdominal CT leads to a marked increase in radiation exposure.

There are several limitations to our study: Firstly, the retrospective study design, secondly, the method we employed to establish diagnostic and therapeutic efficacy. We defined diagnostic efficacy of thoracic CT imaging by calculating the proportion of CTs with pathologic thoracic findings visible only above diaphragmatic dome out of all performed CTs, based on the definition of Fryback and Thornbury, who defined “diagnostic accuracy efficacy” as the yield of abnormal or normal diagnoses in a case series as well as diagnostic accuracy, sensitivity, and specificity, which was not evaluated in our study. On the other hand, they defined “diagnostic thinking efficacy” focusing on whether the information produces change in the referring physician’s diagnostic thinking. Probably, diagnostic thinking efficacy is higher than our defined diagnostic efficacy, since the information, that no thoracic pathologies are present, can also lead to a change in diagnostic thinking. Since this is very difficult to measure, we decided to use the abovementioned definition. We also tried to counter this limitation by assessing the even more relevant therapeutic efficacy, which assesses effects on patient management. Another limitation is the heterogeneity of our study population. The common denominator is infectious complications after surgery for a primary abdominopelvic pathology and the thoracic CT in addition to abdominal CT. We chose to accept this degree of heterogeneity to gain the advantage of including a large group of patients.

## Conclusions

Thoracic CT to identify an infectious focus in ICU patients after abdominopelvic surgery led to the detection of previously unknown pathologies in around 16%. For the purpose of identifying an infectious focus, the diagnostic efficacy was 3.5%, and overall, changes in patient management were only made in one case (0.7%). Thus, the widespread use of thoracic CT in this patient population should be critically evaluated on an individual level, particularly since many relevant thoracic pathologies are readily visible on abdominal CT.

## Data Availability

The datasets used and analyzed during the current study are available from the corresponding author on reasonable request.

## Abbreviations

ICU: Intensive care unit
CXR: Chest radiography

## References

[1] Herwaldt LA, Cullen JJ, Scholz D, French P, Zimmerman MB, Pfaller MA (2006). A prospective study of outcomes, healthcare resource utilization, and costs associated with postoperative nosocomial infections. Infect Control Hospital Epidemiol.

[2] Fleisher LA, Linde-Zwirble WT (2014). Incidence, outcome, and attributable resource use associated with pulmonary and cardiac complications after major small and large bowel procedures. Perioper Med (Lond).

[3] Reeves N, Torkington J (2022). Prevention of surgical site infections. Surg Infect (Larchmt).

[4] Plaeke P, De Man JG, Coenen S, Jorens PG, De Winter BY, Hubens G (2020). Clinical- and surgery-specific risk factors for post-operative sepsis: a systematic review and meta-analysis of over 30 million patients. Surg Today.

[5] Hecker A, Reichert M, Reuß CJ, Schmoch T, Riedel JG, Schneck E (2019). Intra-abdominal sepsis: new definitions and current clinical standards. Langenbecks Arch Surg.

[6] Chidambaranath R, Rajebhosale R, Thomas P (2021). EP.TH.756Post-operative sepsis: Is CT reliable in diagnosing the cause of post-operative sepsis?. Br J Surg.

[7] Laroia AT, Donnelly EF, Henry TS, Berry MF, Boiselle PM (2021). ACR Appropriateness Criteria® Intensive Care Unit Patients. J Am Coll Radiol.

[8] Ganapathy A, Adhikari NK, Spiegelman J, Scales DC (2012). Routine chest x-rays in intensive care units: a systematic review and meta-analysis. Crit Care.

[9] Palazzetti V, Gasparri E, Gambini C, Sollazzo S, Saric S, Salvolini L (2013). Chest radiography in intensive care: an irreplaceable survey?. Radiol med.

[10] Lohan R (2019). Imaging of ICU Patients. Thoracic Imaging.

[11] Smith-Bindman R, Miglioretti DL, Johnson E, Lee C, Feigelson HS, Flynn M (2012). Use of diagnostic imaging studies and associated radiation exposure for patients enrolled in large integrated health care systems, 1996–2010. JAMA.

[12] Kwee TC, Dijkstra H, Knapen DG, Vries EGE de, Yakar D. Which patients are prone to undergo disproportionate recurrent CT imaging and should we worry? Eur J Radiol. 2020;125:108898. 10.1016/j.ejrad.2020.108898 32088659

[13] Pola A, Corbella D, Righini A, Torresin A, Colombo PE, Vismara L (2018). Computed tomography use in a large Italian region: trend analysis 2004–2014 of emergency and outpatient CT examinations in children and adults. Eur Radiol.

[14] Dorenbeck U, Bein T, Strotzer M, Geissler A, Feuerbach S (2002). Thoracic computed tomography in intensive care patients - evaluation of clinical usefulness. Anasthesiol Intensivmed Notfallmed Schmerzther.

[15] Miller WT, Tino G, Friedburg JS (1998). Thoracic CT in the intensive care unit: assessment of clinical usefulness. Radiol Radiolog Soc NAm.

[16] Fryback DG, Thornbury JR (1991). The Efficacy of Diagnostic Imaging. Med Decis Making.

[17] Huda W, Ogden KM, Khorasani MR (2008). Converting dose-length product to effective dose at CT. Radiology.

[18] Schramm D, Bach AG, Meyer HJ, Surov A (2016). Thrombotic events as incidental finding on computed tomography in intensive care unit patients. Thromb Res.

[19] Schegerer A, Loose R, Heuser LJ, Brix G (2019). Diagnostic reference levels for diagnostic and interventional X-Ray procedures in Germany: update and handling. Fortschr Röntgenstr.

